# Management of Horizontal Root Fracture in the Middle Third via Intraradicular Splinting Using a Fiber Post

**DOI:** 10.1155/2016/9684035

**Published:** 2016-01-24

**Authors:** Ishani Karhade, Meenal N. Gulve

**Affiliations:** Usha Kiran Bunglow, Anand Nagar, Behind Muktidham, Opp Maharshi Hospital, Nashik Road, Nashik, Maharashtra 422101, India

## Abstract

Radicular fractures in permanent teeth are uncommon injuries and account for only 0.5–7% of dental traumas. These fractures commonly result from a horizontal impact and are transverse to oblique in direction. Their incidence is more in the middle third of the root than at the apical and cervical thirds. This paper describes a case of complicated crown fracture of maxillary incisors along with horizontal root fracture at the middle third of maxillary right central and lateral incisor. The fractured root fragments of the upper right central and lateral incisor were united with the help of a glass fiber post after receiving an endodontic treatment. The other two incisors were treated endodontically followed by post endodontic restorations. Eventually the four incisors were restored with porcelain fused to metal crowns. A one-year follow-up revealed a well stabilized assembly of the root fragments and the post.

## 1. Introduction

Among all dental traumatic injuries, root fractures account for only 0.5–7% [1–3]. Root fracture is defined as “fracture involving dentin, cement and pulp” [1]. Horizontal root fracture most commonly occurs in the middle third of the root and very rarely in the coronal and apical third [4]. Maxillary central incisors are more prone to traumatic injuries (approximately 68%) probably due to their position in the dental arch. The next in line are the maxillary lateral incisors (27%) followed by mandibular incisors (5%) [5, 6].

Root fracture occurs as a consequence of an impact force on the top of the root and frontal forces affect the compression zone labially and lingually/palatally, thus dividing the root into coronal and apical fragments. This can traumatize the supporting periodontal tissues eventually leading to displacement of the root fragments [7]. Proper clinical and radiographic examination is required for correctly diagnosing root fracture. A clinician must check for mobility of the coronal fragment and the pulp vitality. Radiographically, a radiolucent line is seen separating the apical and coronal fragments [8, 9]. Two or three radiographs taken at different angulations may be sometimes needed to detect the angle of fracture.

The management of horizontal root fracture depends on the location of the fracture and mobility and the vitality of the tooth. Fractures in the apical third usually display no mobility and generally do not require any treatment. Root fracture at the cervical third often requires extraction. When the coronal fragment shows severe mobility, there is no other option but extraction.

Root fractures at the middle third have favourable prognosis. When the coronal fragment is displaced, the initial treatment should be repositioning the fragments, followed by stabilization to allow healing of the surrounding periodontal tissues [1]. With the ever-increasing demand for aesthetics, tooth coloured fiber posts have been introduced, which can be used as a medium to retain the two fractured root fragments in conjunction with bonding agents and composite resins. This report thereby describes a case of horizontal root fracture with displaced fragments, which were united with the help of glass fiber posts followed by core build-up and coronal coverage.

## 2. Case Report

**Figure 1:**
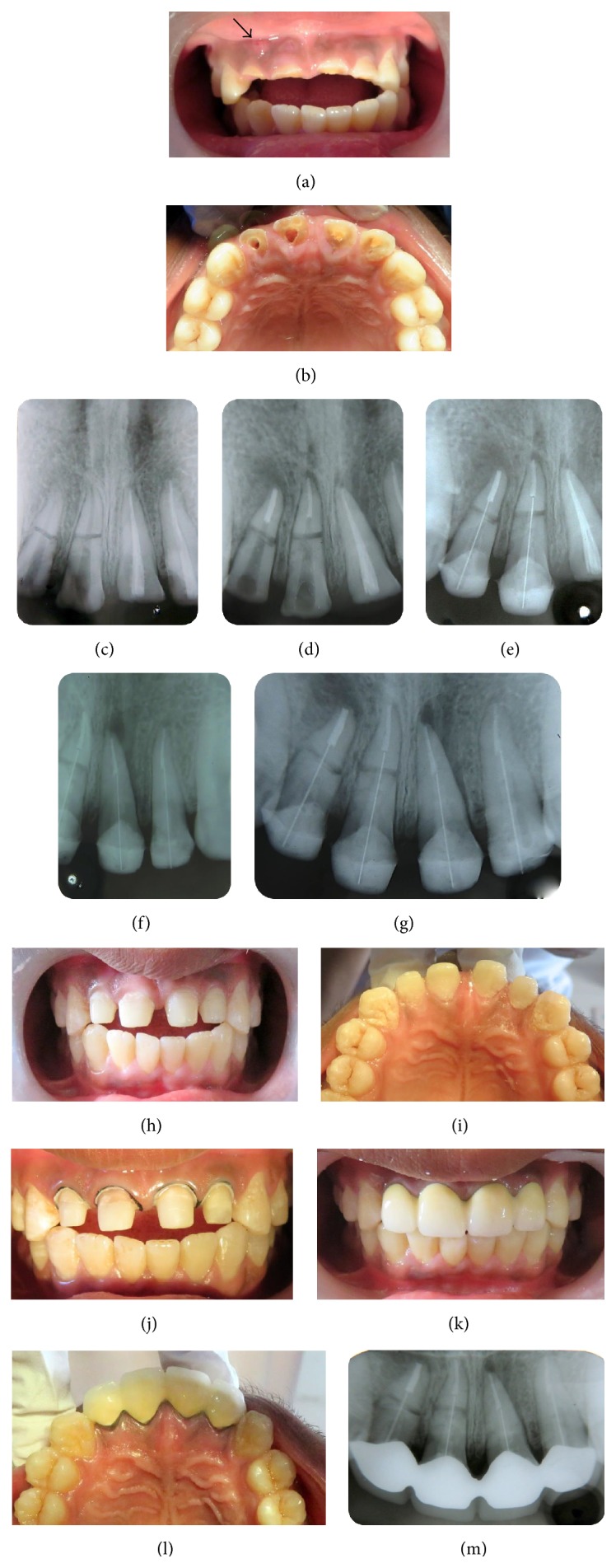
Procedural steps. (a) Preoperative clinical photograph (labial view) demonstrating complicated crown fractures of upper central and lateral incisors and the presence of a sinus tract on labial gingiva. (b) Preoperative palatal view. (c) Preoperative radiograph showing horizontal root fracture at the middle third of upper right central and lateral incisors. (d) Sectional obturation using gutta percha cone and AH plus sealer followed by post space preparation. (e) Core build-up following cementation of post. (f) Post and core treatment of maxillary left central and lateral incisors. (g) Radiograph illustrating post and core treatment of the four upper incisors. (h) Clinical photograph after post and core treatment of upper incisors (labial view). (i) Palatal view. (j) Tooth preparation to receive coronal coverage. (k) Cementation of porcelain fused to metal crowns (labial view). (l) Palatal view. (m) Postoperative radiograph after cementation of the crowns.

A 19-year-old male patient reported to the department of conservative dentistry and endodontics following trauma to the maxillary anterior region due to a road accident around 20 days back. He complained of fractured maxillary incisors and presented a desire to get them restored in order to have an aesthetic smile. Clinical examination of the patient revealed complicated crown fracture of the upper incisors. An intraoral swelling with a sinus tract was present on the labial gingiva between upper right central and lateral incisor (Figures 1(a) and 1(b)). A radiograph of the maxillary anterior region illustrated horizontal root fracture at the middle third of the upper right central and lateral incisors (Figure 1(c)) but clinically no mobility of the coronal fragment was evident. On the other hand the roots of the upper left central and lateral incisors were intact and endodontic treatment had been done for the upper left central and lateral incisors (Figure 1(c)).

After explaining the treatment plan to the patient and obtaining his consent, endodontic treatment with the upper right central and lateral incisors was initiated. The working length was correctly determined and canals were cleaned, shaped using K files in a step-back manner to an apical file size #60. The remainder of the canals were shaped to obtain a uniform taper from apex towards coronal. An interappointment calcium hydroxide dressing was given and the patient was recalled after 7 days.

On the second visit, when the swelling had resolved, root canals were sectionally obturated with gutta percha cone and AH plus sealer. A gutta percha cone of the same size of the prepared root canal (size #60, with a taper of 2%) was selected and tried into the canal to obtain a snug fit. It was then cut to obtain a section which would be 2-3 mm short of the apical fragment of the root. A suitable plugger which loosely fits 2-3 mm short of the apical root fragment was selected and a stopper was set at this length. The sectioned gutta percha was then coated with AH plus sealer. One end of gutta percha was mounted to a heated plugger and then carried into the canal to the desired length. After this, gutta percha was disengaged from the plugger by slightly rotating the plugger in anticlockwise direction (Figure 1(d)).

After 5 days of recall, the tooth was asymptomatic and the sinus tract healed. Next, glass fiber posts were used to retain the fractured root fragments. Appropriate glass fiber posts were tried into the canals, adjusted to the desired length until they just passively touched the apical gutta percha. Root canals were etched with 37% phosphoric acid gel and dried with paper points. The fiber posts were luted with dual cure resin cement, inserted into the canal without applying any pressure, and then light cured for 40 seconds. The benefit of higher viscosity of the cement in absence of pressure is that it reduces the flow of the resin. Also, the resin was used cautiously only in the amount necessary to achieve a desirable bond between the post and the dentin. Only the post was luted with the cement. Coating the root canal walls with resin cement was precluded to prevent the flow of excess cement laterally between the root fragments.

These fiber posts served as an intraradicular splint, stabilizing the fractured fragments in position. Glass fiber-reinforced posts were used as they exhibit high fatigue strength and high tensile strength and have a modulus of elasticity closer to dentin. Composite cores were built over the posts (Figure 1(e)). The other two incisors (maxillary left central and lateral incisors) received a similar post and core treatment (Figures 1(f)–1(i)). The teeth were then restored with full-coverage porcelain fused to metal crowns (Figures 1(j)–1(m)).

**Figure 2:**
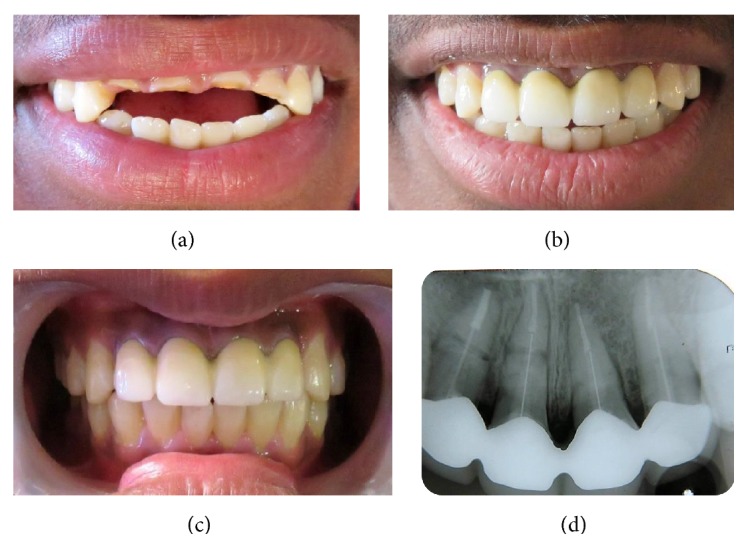
(a) Preoperative smile of the patient. (b) Postoperative smile. (c) Clinical photograph after one year of follow-up. (d) Postoperative radiograph after one year of follow-up.

The patient was reevaluated on a regular basis. After 12 months of recall, the patient presented with aesthetically pleasing results and sound periodontium and the fractured root fragments were well retained with the aid of a post (Figures 2(c) and 2(d)).

## 3. Discussion

Root fracture can be a consequence of dental trauma causing a complex injury to the cementum, dentin, pulp, and the periodontal tissues [10]. Such injuries can occur due to road accidents, violence, sport injuries, and so forth. Maintaining “the physiological and functional integrity” is the main goal while treating traumatized teeth.

Four types of conservative endodontic treatment that have been commonly described [11, 12] are (i) cleansing and gutta percha (GP) filling of the root canal of the coronal fragment only; (ii) cleansing and filling of the root canal in both fragments; (iii) cleansing and GP filling of the root canal of the coronal fragment and surgical removal of the apical fragment; and (iv) treatment of the root canal with calcium hydroxide followed by filling with GP. Recently, different types of post materials have been introduced into the dental practice such as carbon fiber, quartz, and glass fiber. The fiber posts offer several advantages such as a suitable elastic modulus, aesthetics, good bonding between post and cement, lower chair side time, and minimal tissue removal.

In the present case report, only the apical fragment was endodontically treated and the two fragments were splinted using a glass fiber post to act as an intraradicular splint. After luting the post with resin cement, it was inserted into the root canal passively, without applying pressure. This is because higher viscosity of the resin in absence of pressure reduces its flow. This may prevent the resin from flowing between the root fragments [13].

The conventional metal posts have a high modulus of elasticity [14], whereas the fiber- reinforced posts have a modulus similar to that of the dentin, minimizing the risk of root fractures [15]. According to Gurtu and Singhal [16], the use of a post ensures support and stability to the tooth. It also helps to retain the root fragments by radicular anchoring thereby strengthening the restoration complex which is subjected to tangential stresses. Further, it creates a monoblock between the post, reconstructive material, cement, and the tooth [17]. Post placement, in addition to bonding, provides retention via a friction bond and assists in preventing dislodgement to nonaxial forces [18]. Thus, light-transmitting fiber posts have been widely used to functionally and aesthetically restore the compromised root filled teeth. Teeth, which earlier would have been condemned to extraction, could now be strengthened by a sufficiently thick lining of intraradicular reinforcing composite, thereby salvaging them for continued function [19].

Linkow [20] proposed that, by inserting a post through the tooth, deep into the bone and cementing the intradental part to the root canal walls, the fulcrum of movement of a loose tooth is moved deeper into the jaw, the support in the bone is increased, and thus the mobility of the tooth is lessened.

Luting agents including zinc phosphate, zinc polycarboxylate, glass ionomer, and resin modified glass ionomer have been used traditionally. They had disadvantages such as solubility in oral fluids, especially in the presence of acid and lack of true adhesion. Resin modified glass ionomers exhibit hygroscopic expansion and hence their use declined. Nowadays adhesive resin cement has been advocated for cementation of post because it bonds the post to the tooth structure. There is not only chemical adhesion but also micromechanical bonding. However, a disadvantage of this resin cement is its technique sensitivity. Also, the bonding to root canal dentin could be compromised due to the use of various irrigants and eugenol based sealers. Eugenol can prevent or stop the polymerization reaction and can also interfere with bonding [21].

Midroot fractures can heal by different mechanisms. Andreasen and Hjörting-Hansen described four types of healing sequelae [22]: (1) healing with calcified tissue, (2) healing with interproximal connective tissue, (3) healing with interproximal bone and connective tissue, and (4) interproximal inflammatory tissue without healing.

A long-term follow-up is required to check for any possible pathological alterations. Follow-up of this case after one year showed promising results with clinically pleasing aesthetics and radiographic healing with calcified tissue, the fractured line discernible but fragments well stabilized.

## 4. Conclusion

“Preservation of natural dentition and restoration of the oral cavity to a normal functional state” is the primary goal in dentistry. The main aim of treating fractured elements is to keep the tooth steady and maintain its position in the dental arch whenever possible. The improvement in bonding agents and restorative resins and the availability of newer materials like the fiber posts and dual cure resin cement have provided clinicians with different modalities for successfully managing root fractures. Intraradicular splinting using fiber posts can be a good alternative for managing midroot fractures and reestablishing the aesthetics and functional needs of the patients.
